# Diagnostic Accuracy and Confidence in Management of Forearm and Hand Fractures Among Foundation Doctors in the Accident and Emergency Department: Survey Study

**DOI:** 10.2196/45820

**Published:** 2023-08-18

**Authors:** Ben Gompels, Tobin Rusby, Richard Limb, Peter Ralte

**Affiliations:** 1 Cambridge University Cambridge United Kingdom; 2 Wirral University Teaching Hospital Liverpool United Kingdom; 3 Aintree University Teaching Hospital Liverpool United Kingdom

**Keywords:** education, diagnostic accuracy, doctor, fracture, x-ray, radiograph, diagnostic error, patient safety

## Abstract

**Background:**

Accurate interpretation of radiographs is crucial for junior doctors in the accident and emergency (A&E) department (the emergency medicine department). However, it remains a significant challenge and a leading cause of diagnostic errors.

**Objective:**

This study aimed to evaluate the accuracy and confidence of foundation doctors (doctors within their first 2 years of qualifying) in correctly interpreting and managing forearm and hand fractures on plain radiographs.

**Methods:**

A total of 42 foundation doctors with less than 2 years of experience and no prior emergency medicine training who worked in a large district general hospital participated in a web-based questionnaire. The questionnaire consisted of 3 case studies: distal radius fracture, scaphoid fracture, and a normal radiograph. Respondents were required to identify the presence or absence of a fracture, determine the fracture location, suggest appropriate management, and rate their confidence on a Likert scale.

**Results:**

Overall, 48% (61/126) of respondents accurately identified the presence and location of fractures. The correct management option was chosen by 64% (81/126) of respondents. The median diagnostic confidence score was 4 of 10, with a mean diagnostic certainty of 4.4 of 10. Notably, respondents exhibited a significantly lower confidence score for the normal radiograph compared to the distal radius fracture radiograph (*P*=.01).

**Conclusions:**

This study reveals diagnostic uncertainty among foundation doctors in interpreting plain radiographs, with a notable inclination toward overdiagnosing fractures. The findings emphasize the need for close supervision and senior support to mitigate diagnostic errors. Further training and educational interventions are warranted to improve the accuracy and confidence of junior doctors in radiographic interpretation. This study has several limitations, including a small sample size and reliance on self-reported data. The findings may not be generalizable to other health care settings or specialties. Future research should aim for larger, more diverse samples and explore the impact of specific educational interventions on diagnostic accuracy and confidence.

## Introduction

Interpretation of radiographs presents a challenge for junior doctors and is one of the main causes of diagnostic error in a hospital environment [1]. One large scale study over 4 years at an accident and emergency (A&E) department (the emergency medicine department) cited that almost 80% of diagnostic errors over this period were due to misread radiographs [2]. Additionally, fractures have been cited as one of the most common missed diagnoses [3]. Missed or delayed diagnoses have a significant impact on patient safety and standards of care. In the National Health Service (NHS), the A&E department is often one of the first environments where junior doctors are personally responsible for the discharge of patients from hospital. This can pose both a diagnostic challenge and place medico-legal responsibility on health professionals who have limited experience.

Studies indicate that the accuracy of junior doctors in correctly making diagnoses from radiographs varies widely [2]. Factors can be divided into clinician- and non–clinician-related factors. Clinician-specific factors include experience, level of training, and exposure to previous trauma [4]. Non–clinician-related factors have been shown to influence accurate radiograph interpretation, including time of day and location of fracture [5,6]. Junior doctors tend to have lower accuracy compared to more experienced clinicians and radiologists and lower confidence in their diagnoses. This has been attributed in part to the limited teaching on radiograph interpretation at medical schools [7].

Previous literature has highlighted that the supervision of junior trainees in the interpretation of radiographs is paramount [8]. One of the main causes of missed diagnoses in the A&E department for trauma is missed fractures; factors contributing to this include fatigue, inexperience, and extended working hours [2,3]. Studies indicate a relatively high error rate in junior doctors’ ability to diagnose abnormalities on radiographs, though the overall incidence of missed diagnosis is lower, with some studies suggesting a figure of 3% [9].

In this study, the decision was made to focus on common upper extremity fractures. In considering which fractures to choose, the authors were influenced by the fact that the most common fracture seen in adult trauma is of the distal radius [10]. Similarly, scaphoid fractures are the most common carpal fracture and account for between 2% to 7% of all fractures [11].

The current literature has evaluated error rate and accuracy of junior trainees (trainees within their first 2 years of qualifying), but few studies have looked at the confidence of trainees in their own diagnosis and management of upper limb fractures. This study aimed to look at the diagnostic accuracy and confidence of foundation trainees (doctors within their first 2 years of qualifying) in A&E in interpreting common upper limb fracture patterns.

## Methods

### Study Design

A survey was conducted among foundation doctors who were in their first 2 years since graduating and were in the initial 3 weeks of their A&E rotation. This was distributed to 2 different cohorts of doctors over the period of 8 months. The survey was conducted on the web and comprised 3 case studies, each presenting a clinical vignette along with plain radiographs of the hand and wrist. The participants were required to determine if a fracture was present, specify the location of the fracture, suggest the appropriate management approach, and rate their subjective confidence in their diagnoses on a Likert scale of 1 to 10. In case of uncertainty, participants were allowed to select “unsure” as their answer. The survey also inquired whether the respondents had previously completed a rotation in trauma and orthopedics before their A&E placement. The data were anonymized and stored securely on a password-protected web-based database.

The management options for each radiograph were based on acute A&E protocols and categorized into nonoperative reduction with or without plaster or splint, operative intervention, referral to orthopedics, or no definitive management required. The cases, radiographs, and management were reviewed and checked for accuracy by the senior author (PR), a trauma and orthopedic consultant. The correct management for each scenario was determined based on the peer-reviewed guidelines from BMJ Best Practice regarding acute fracture management [12].

### Ethical Considerations

Respondents were informed that their responses were being collected as part of an educational study into fracture recognition and diagnostic confidence and that their responses were anonymized. No payment or compensation was made for completion of the survey.

### Cases

Case 1 consists of an anterior posterior (AP) and lateral radiograph of a scaphoid fracture. The accompanying clinical vignette states: “A young male presents to A&E with wrist pain following a fall onto an outstretched hand, this is a closed and neurovascularly intact injury.”

Case 2 consists of an AP and lateral radiograph of a distal radius fracture. The accompanying clinical vignette states: “A middle aged female presents to A&E with wrist pain with a fall onto an outstretched hand, this is a closed and neurovascularly intact injury.”

Case 3 consists of an AP and lateral radiograph with no fracture. The accompanying clinical vignette states: “A middle aged female presents to A&E with wrist pain following a fall onto an outstretched hand, this is a closed and neurovascularly intact injury.”

All images for the plain radiographs were sourced under the Creative Commons license from a radiography reference website [13-15].

## Results

### Overview

A total of 42 responses to the web-based survey were collected. The data obtained from these responses have been compiled and summarized in Table 1, presenting the following information for each case: number and percentage of respondents who believed there was a fracture; number and percentage of respondents who accurately identified the presence of a fracture (if one was indeed present); and number and percentage of respondents who correctly identified whether the management of the fracture required an operation.

**Table 1:** Analysis of results; percentages are of total respondents (N=42).

Case (diagnosis)		Identified whether fracture present	Location of fracture	Management of fracture	Confidence in diagnosis			
					Median score		Mean score	
**Case 1 (scaphoid fracture)^a^, n (%)**						4		4.5
	Correct	29 (69)	17 (40)	31 (74)				
	Incorrect	7 (17)	14 (33)	3 (7)				
	Unsure	6 (14)	3 (7)	8 (19)				
	Missed	N/A^b^	8 (19)	N/A				
**Case 2 (distal radius fracture)^c^, n (%)**						5		5
	Correct	35 (83)	30 (71)	31 (74)				
	Incorrect	2 (5)	6 (14)	4 (10)				
	Unsure	5 (12)	4 (10)	7 (17)				
	Missed	N/A	2 (5)	N/A				
**Case 3 (no fracture), n (%)**						3.5		3.7
	Correct	14 (33)	14 (33)	19 (45)				
	Incorrect	12 (29)	16 (38)	3 (7)				
	Unsure	16 (38)	12 (29)	20 (48)				
**Total, n**						4		4.4
	Correct	62	48	64				
	Incorrect	17	29	8				
	Unsure	21	15	28				
	Missed	N/A	12	N/A				

^a^Difference in Likert score between scaphoid and no fracture: *P*=.08 (Mann-Whitney test).

^b^N/A: not applicable.

^c^Difference in Likert score between radius and no fracture: *P*=.01 (Mann-Whitney test).

### Fracture Presence and Location

Across all 3 case studies, the presence of a fracture was correctly identified by 62% (78/126) of respondents. However, only 48% (61/126) of respondents accurately pinpointed the location of the fracture. In 12% (10/84) of cases, fractures were entirely missed, while the correct management option was chosen in 64% (81/126) of cases.

When examining the specific fractures, 40% (17/42) of respondents correctly identified the presence and location of the scaphoid fracture, compared to 71% (30/42) for the distal radius fracture. Approximately 33% (14/42) of respondents correctly determined that the final radiograph had no fracture present.

### Management

Across all 3 cases, the correct management option was selected by 64% (81/126) of respondents. For both distal radius fractures and scaphoid fractures, the correct management options were selected by 74% (31/42) of respondents in each case.

### Confidence in Diagnosis

The overall median diagnostic confidence score across all 3 scenarios was 4 of 10 (with 10 indicating full confidence), with a mean diagnostic certainty of 4.4 of 10. In the case scenario where a normal radiograph showed no fracture, respondents displayed lower mean and median confidence scores compared to the radiographs with fractures: 3.7 for the scaphoid fracture and 5 for the distal radius fracture. There was a statistically significant difference in confidence scores between respondents for the distal radius fracture and the normal radiograph (Mann-Whitney test; *P*=.01).

## Discussion

### Principal Results

This study aimed to look at the diagnostic accuracy and confidence of foundation trainees in the A&E department in identifying common upper limb fractures and managing them. Overall, 48% (61/124) of respondents accurately identified the presence and location of fractures. The correct management option was chosen by 64% (81/126) of respondents. The median diagnostic confidence score was 4 of 10, with a mean diagnostic certainty of 4.4 of 10. Notably, respondents exhibited a significantly lower confidence score for the normal radiograph compared to the distal radius fracture radiograph (*P*=.01).

### Identification of the Correct Fracture Location Varied Between Radiographs

The ability to accurately locate fractures varied among respondents, with only 40% (17/42) correctly identifying the location of the scaphoid fracture, while 71% (30/42) accurately identified the presence and location of the distal radius fracture. It is worth noting that in clinical practice, radiographs have limitations in detecting scaphoid fractures, especially in the early stages following an injury, with a reported false negative rate ranging from 20% to 54% [16].

The proximal pole of the scaphoid is susceptible to posttraumatic avascular necrosis due to its restricted blood supply, in contrast to the distal pole [17]. Consequently, scaphoid fracture diagnoses are often missed, and this can lead to litigation in the NHS, highlighting their significance as a common cause of legal disputes [18].

These findings emphasize the challenges associated with accurately identifying and managing scaphoid fractures, underscoring the need for improved diagnostic techniques and strategies to mitigate the risk of missed diagnoses and potential complications. Enhanced awareness, thorough clinical examination, and consideration of additional imaging modalities may help improve the detection and proper management of scaphoid fractures, ultimately improving patient outcomes and reducing the associated medico-legal consequences.

### Diagnostic Confidence of Respondents

The diagnostic confidence of junior doctors was found to be higher when detecting fractures compared to ruling them out. On the Likert scale, the confidence scores for the normal radiograph were recorded as 3.7, while scores of 4 and 5 were obtained for the scaphoid fracture and radial fracture, respectively. This discrepancy in confidence levels may be attributed to the limited experience of the respondents in interpreting normal radiographs.

Notably, less than one third (12/42, 29%) of the surveyed foundation doctors had previous exposure to orthopedics placements, which serve as valuable learning environments for the interpretation of plain films.

These findings highlight the impact of clinical exposure and specific training in developing the necessary skills for accurate interpretation of radiographic images. The lower confidence in identifying normal radiographs underscores the need for increased exposure and educational interventions to familiarize junior doctors with a wide range of radiological presentations, including those without apparent pathology.

### Management

The correct management option was identified in 74% (31/42) of radiographs where a fracture was present, and the overall accuracy across all 3 case studies was 64% (81/126). It is important to note that the indications for operative management of distal radius fractures and scaphoid fractures require specialized orthopedic knowledge and are typically managed in outpatient settings. However, it is worth considering that foundation doctors may have limited exposure to the clinical management of orthopedic cases in outpatient settings, which could contribute to the observed finding.

Given the complex nature of determining the appropriate management approach for fractures, further emphasis on orthopedic outpatient clinical exposure and educational interventions during training could help improve the decision-making skills of junior doctors. Enhancing their understanding of the indications for operative management and the nuances associated with different fracture types would better equip them to make informed management decisions.

### Comparison With Prior Work

Accurate interpretation of plain radiographs and appropriate management decisions are critical in the emergency department and for ongoing patient care. Prior studies have emphasized the positive impact of prompt diagnosis of upper limb injuries on quality of life and successful return to work [19].

However, studies have demonstrated significant variability in the diagnostic accuracy of junior doctors when interpreting trauma radiographs. For instance, a study conducted in the A&E setting reported an approximate incidence of misdiagnosis of 1.8% for sprained wrists [20]. A large-scale study focusing on the A&E department revealed an overall accuracy range of 59% to 73% for radiographs of the wrist and hand [1]. In our study, the overall accuracy was 48% (61/126), falling below this range.

It is crucial to acknowledge that a comprehensive clinical examination and detailed history-taking play a vital role in achieving accurate diagnoses by correlating symptoms with radiological findings. For example, tenderness within the anatomical snuffbox has been identified as the single most specific clinical marker for scaphoid fracture [21]. Additionally, thorough history-taking and clinical context have been shown to enhance the accuracy of radiograph interpretation, such as in the case of chest films [4]. It is plausible that the limited accuracy observed among respondents in our study may be attributed to the absence of such clinical information. Our findings highlight the importance of integrating focused clinical examination and history-taking alongside radiographic interpretation to improve diagnostic accuracy.

### Limitations

This study had several limitations that should be acknowledged.

First, the reliance solely on radiographic findings without the inclusion of clinical examination and history-taking may have constrained the respondents’ ability to accurately diagnose and manage the fractures presented. Incorporating these additional diagnostic tools could have provided a more comprehensive assessment.

Another limitation is the small sample size of 42 respondents, which may restrict the generalizability and interpretation of the findings. The study only involved a single cohort of junior doctors, potentially overlooking variations that could exist among medical graduates from different years or backgrounds.

It is important to consider these limitations when interpreting the results, and future studies should aim to address them by incorporating a larger and more diverse sample size, as well as a comprehensive clinical assessment alongside radiographic findings.

### Conclusion

In conclusion, this study of a cohort of foundation doctors embarking on their A&E rotation highlights the variability in accurately identifying the location of fractures across different radiographs. Interestingly, the diagnostic confidence of junior doctors was found to be higher when detecting fractures compared to ruling them out. To mitigate potential diagnostic errors and ensure optimal management, it is imperative to provide close supervision and senior support. Our findings underscore the importance of implementing further teaching interventions aimed at enhancing the clinical accuracy and confidence of junior doctors in fracture diagnosis. Future research should focus on exploring targeted educational strategies that facilitate the development of robust diagnostic skills among junior doctors.

## Abbreviations

A&E: accident and emergency department
AP: anterior posterior
NHS: National Health Service

## Data Availability

The data sets generated during and/or analyzed during this study are available from the corresponding author on reasonable request
